# Protein Clusters on the T Cell Surface May Suppress Spurious Early Signaling Events

**DOI:** 10.1371/journal.pone.0044444

**Published:** 2012-09-04

**Authors:** Woo Chung, Steven M. Abel, Arup K. Chakraborty

**Affiliations:** 1 Department of Chemical Engineering, Massachusetts Institute of Technology, Cambridge, Massachusetts, United States of America; 2 Department of Chemistry, Massachusetts Institute of Technology, Cambridge, Massachusetts, United States of America; 3 Department of Biological Engineering, Massachusetts Institute of Technology, Cambridge, Massachusetts, United States of America; 4 Ragon Institute of MGH, Massachusetts Institute of Technology, Cambridge, Massachusetts, United States of America; 5 Harvard, Charlestown, Massachusetts, United States of America; University of Oslo, Norway

## Abstract

T cells play an important role in the adaptive immune system, quickly activating effector functions in response to small numbers of antigenic peptides but rarely activating in response to constant interaction with most endogenous peptides. Emerging experimental evidence suggests that key membrane-bound signaling proteins such as the T cell receptor and the adaptor protein Lat are spatially organized into small clusters on the T cell membrane. We use spatially resolved, stochastic computer simulations to study how the inhomogeneous distribution of molecules affects the portion of the T cell signaling network in which the kinase ZAP-70, originating in T cell receptor clusters, phosphorylates Lat. To gain insight into the effects of protein clustering, we compare the signaling response from membranes with clustered proteins to the signaling response from membranes with homogeneously distributed proteins. Given a fixed amount of ZAP-70 (a proxy for degree of TCR stimulation) that must diffuse into contact with Lat molecules, the spatially homogeneous system responds faster and results in higher levels of phosphorylated Lat. Analysis of the spatial distribution of proteins demonstrates that, in the homogeneous system, nearest ZAP-70 and Lat proteins are closer on average and fewer Lat molecules share the same closest ZAP-70 molecule, leading to the faster response time. The results presented here suggest that spatial clustering of proteins on the T cell membrane may suppress the propagation of signal from ZAP-70 to Lat, thus providing a regulatory mechanism by which T cells suppress transient, spurious signals induced by stimulation of T cell receptors by endogenous peptides. Because this suppression of spurious signals may occur at a cost to sensitivity, we discuss recent experimental results suggesting other potential mechanisms by which ZAP-70 and Lat may interact to initiate T cell activation.

## Introduction

Nearly all vertebrates, including humans, are equipped with an adaptive immune system that can respond to diverse and previously unencountered pathogens. T lymphocytes (T cells), the primary orchestrators of adaptive immunity, are equipped with molecular features that enable them to distinguish molecular markers of pathogens from “self.” The identification of foreign pathogens is accomplished largely through the T cell receptor (TCR), which is a transmembrane protein that interacts with short peptide fragments complexed with host proteins known as major-histocompatibility complexes (MHCs) displayed on other cells. If the TCR and peptide-MHC (pMHC) complex bind sufficiently strongly, then the T cell can become activated through a network of biochemical reactions that originate with the TCR and ultimately change the gene transcription program of the cell [1]. T cell activation can be elicited by a small number of antigenic peptides.

While a T cell can be activated upon encountering a few antigenic pMHC, it is important to note that T cells nearly constantly encounter endogenous peptide fragments bound to MHC, yet rarely activate upon numerous such interactions. This feature of cell signaling is essential for the integrity of the immune system, as frequent activation of T cells against self peptides could lead to uncontrolled proliferation of T cells, a hallmark of autoimmunity. Although the key biochemical components of the early T cell signaling network are known, in spite of advances [2], [3], [4], how the network of biochemical reactions yields a fast and specific response against small numbers of antigenic pMHC remains an open question.

The earliest step in T cell signaling through the TCR involves the cytoplasmic portion of the TCR complex, which contains several immunoreceptor tyrosine-based activation motifs (ITAMs). These motifs can be phosphorylated by the tyrosine kinase Lck when the TCR is bound to a peptide-MHC complex. Once phosphorylated, the ITAMs act as binding domains for the kinase ZAP-70 [4], [5], [6], [7], [8]. When recruited, ZAP-70 can be phosphorylated by Lck [8], turning it into an active tyrosine kinase. Active ZAP-70 can phosphorylate several tyrosine residues on Lat (linker of activated T cells), an essential adaptor protein that serves as a docking site for many other proteins involved in downstream signaling. The activation of a T cell is controlled largely by early signaling events that occur quickly, with markers of productive signaling downstream of the TCR detectable on order of 10 seconds after TCR engagement with agonist pMHC ligands [9]. Productive signaling, starting from these earliest events, eventually transforms a T cell into an active state [3].

Both TCRs and Lat molecules are transmembrane proteins, suggesting that it may be important to consider features of the membrane environment when analyzing the spatiotemporal control of T cell signaling. The cell membrane has traditionally been difficult to study experimentally, although emerging experimental evidence suggests that the molecules on resting T cell membrane are not homogeneously distributed, but are spatially organized into clusters of proteins [10], [11], [12], [13], [14], [15], [16], [17]. Recent results by Lillemeier et al. indicate that TCRs and Lat are each clustered into distinct regions on the surface of resting T cells, with the cluster sizes ranging between 30 nm and 700 nm [11], [12]. Additionally, the average distance between neighboring TCR and Lat clusters apparently decreases upon T cell activation [11]. These results differ from the traditional fluid mosaic model which regards the membrane as a lipid bilayer containing a homogeneous distribution of membrane proteins. How the spatial organization of proteins affects signaling is a topic of current interest [18], [19], as spatiotemporal dynamics of proteins may play key roles in regulating cell signaling. In this paper, we use computational methods to examine effects of TCR and Lat clustering on early T cell signaling events using the experimental results of Lillemeier et al. as motivation. We start with a fixed amount of active ZAP-70 as a proxy for TCR stimulation and study a model in which ZAP-70 must diffuse into spatial proximity of Lat to phosphorylate it. We find that the clustering of proteins leads to a slower signaling response (as measured by the amount of phosphorylated Lat) compared with the case in which all proteins are homogeneously distributed. Analysis of the spatial distribution of proteins demonstrates that, in the homogeneous system, neighboring ZAP-70 and Lat proteins are closer on average and fewer Lat molecules share the same neighboring ZAP-70 molecule, leading to faster response times. In the Discussion section, we discuss alternative mechanisms suggested by recent experiments in which protein clusters may enhance TCR signal transduction.

## Methods

### Signaling Network

The methods we use explicitly consider both spatial aspects of the membrane surface and the stochastic nature of diffusion and chemical reactions. We compare systems in which proteins are inhomogeneously distributed in clusters with systems in which key proteins are distributed homogenously. We focus on a small portion of the T cell signaling network in which a kinase (ZAP-70) searches for and modifies a target molecule (Lat) [8], while other proteins (phosphatases such as SHP-1) in the system can inactivate the kinase protein [20]:
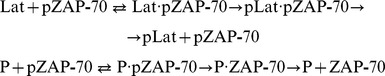



Here, pZAP-70 denotes phosphorylated, active ZAP-70, which acts as a kinase of Lat, and P denotes a phosphatase that binds and dephosphorylates pZAP-70, thus abrogating its catalytic function. This generic scheme is common in cellular signaling networks, so insights gained here are likely to be relevant to other signaling networks with similar motifs. Note that in reality Lat has multiple phosphorylation sites, but we consider only a single modification in order to gain qualitative insight into the effects of clustering.

### Computational Framework

We consider discrete-space, continuous-time dynamics in which two-dimensional space is divided into a square lattice with lattice spacing 0.01 µm. The dynamics are governed by a chemical master equation in which molecules diffuse by hopping to nearest neighbor lattice sites and proteins can react when they co-occupy the same lattice site. Typical reported diffusivities of proteins on the membrane range from 0.01 to 0.5 µm^2^/sec [11], [21], [22]. We use our previously developed Stochastic Simulation Compiler to simulate trajectories consistent with the dynamics [23].

**Figure 1:**
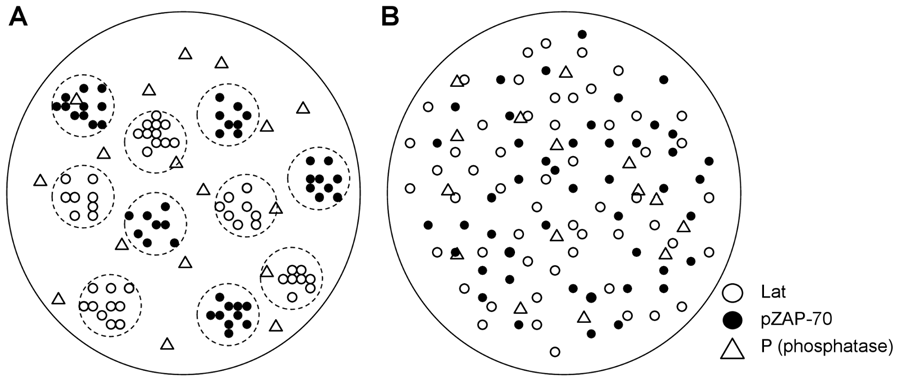
Schematic depiction of the initial distribution of proteins. (**A**) depicts the membrane with clustered proteins, with Lat and pZAP-70 molecules initially located in non-overlapping clusters. Lat molecules are confined to diffuse only within their initial cluster domain, while ZAP-70 molecules can diffuse anywhere on the membrane. (**B**) depicts the homogeneously distributed system, with Lat and pZAP-70 molecules homogeneously distributed initially. In both cases, phosphatase molecules that can deactivate pZAP-70 can diffuse through all domains and are homogeneously distributed initially.

Recent experimental studies suggest that the radius of typical TCR and Lat clusters varies from 35 to 70 nm, and can be up to 300 nm [11], [24], [25]. Additionally, TCR and Lat clusters apparently do not intermix their own components (Lat and TCR molecules) and their domains do not overlap [11]. To model the clustered distribution of proteins, we consider three types of domains: TCR clusters, Lat clusters, and the remaining membrane space not occupied by the clusters (see Fig. 1). Since ZAP-70 is recruited to phosphorylated ITAM regions on the cytoplasmic domain of the TCR complex during early T cell signaling [6], [8], [26], [27], we begin by assuming a fixed amount of active ZAP-70 exists in each TCR cluster and focus on the dynamics of Lat phosphorylation. The fixed amount of ZAP-70 corresponds to ZAP-70 recruited upon TCR stimulation. Thus we do not explicitly consider the dynamics of TCRs and instead focus on slightly downstream signaling reactions. Each simulation is initiated by placing clusters of fixed radius at random positions subject to the constraint that they do not overlap. The entire patch of simulated membrane is circular with a diameter of 2 µm, and reflecting boundary conditions are used. Each cluster is 0.2 µm in diameter. The system size is appropriate for a cell-cell contact region, and we have tested that increasing the system size does not change the results presented below.

For each trajectory, we place a fixed number of ZAP-70 uniformly at random in each TCR cluster and a fixed number of Lat at random in each Lat cluster. Phosphatases are placed uniformly at random over the entire membrane, irrespective of the domain, and are meant to be representative of membrane-proximal cytosolic phosphatases such as SHP-1. Each protein diffuses with a fixed diffusion coefficient, with Lat molecules restricted to diffuse within their initial cluster domain. ZAP-70 and phosphatases can diffuse throughout any domain. In the homogeneous system, there are no cluster domains and all proteins are placed uniformly at random over the entire membrane. For comparison, for each set of conditions with clustered proteins, we consider the homogeneous case with the same number of molecules. The only difference is the initial configuration and the fact that Lat can diffuse anywhere in the homogeneous system. Unless noted otherwise, all simulations are performed using a diffusion coefficient of D = 0.0033 µm^2^/s.

In the system with clustered proteins, we place twenty Lat molecules per cluster and vary the concentration of ZAP-70 molecules per TCR cluster. It is not clear whether stronger antigenic peptides induce higher concentrations of ZAP-70 per cluster. The typical number used was 20 ZAP-70 per cluster (see Tables S1 and S2 for typical parameter values). In addition, since the membrane is two-dimensional, kinetic rate constants are converted to appropriate two-dimensional units (see SI). For every set of conditions and parameters, we generate 500 independent trajectories, which we use to determine the mean and standard deviation of the number of pLat molecules as a function of time. For cases in which proteins are homogeneously distributed, each trajectory is initiated from a random configuration of proteins. For cases in which proteins are clustered, we first generate 20 different random configurations of clusters. For each configuration, we then run 25 independent trajectories in which proteins are initially placed at random positions within their clusters.

## Results

### Pre-existing Clusters Suppress the Phosphorylation of Lat

Intuiting the influence of clusters on the speed and magnitude of downstream signaling is difficult. For example, it is possible that the system with clustered proteins might enhance downstream signals due to the high local concentration of Lat molecules in clusters. Once a few active ZAP-70 molecules enter Lat clusters, they might be able to quickly phosphorylate many Lat molecules through the serial engagement of Lat within the cluster.

**Figure 2:**
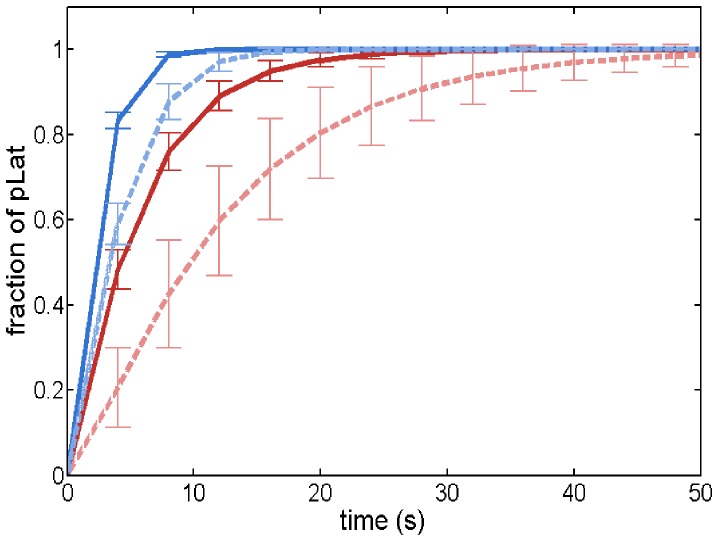
Fraction of phosphorylated Lat as a function of time without phosphatase. The fraction of phosphorylated Lat (pLat) measures the ratio of the number of phosphorylated Lat molecules to the total number of Lat molecules. Solid lines (dark) correspond to the homogeneous system and dashed lines (lighter) correspond to the system with clustered proteins. Dashed red curves have 5 ZAP-70 and 5 Lat clusters, and dashed blue curves have 20 ZAP-70 and 20 Lat clusters. Each cluster initially contains 20 proteins. The homogeneous systems have the same number of molecules but in a homogeneous initial configuration.

We begin by investigating the time required for ZAP-70 molecules to phosphorylate the Lat molecules in the absence of phosphatase. Figure 2 shows that in the homogeneous system, it takes less time to reach a given amount of phosphorylated Lat than in the case with clustered proteins. Hence, in the absence of phosphatase, the system with homogeneously distributed proteins has a faster response than the system with clustered proteins. We explore the physical reasons for this difference in the next section.

**Figure 3:**
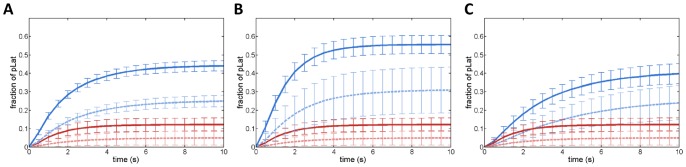
Fraction of phosphorylated Lat as a function of time with phosphatase. Solid lines correspond to the homogeneous system and dashed lines correspond to the system with clustered proteins. (**A**) The effect of increasing the number of clusters at fixed phosphatase concentration (800 phosphatase molecules per π µm^2^). Dashed red curves have 5 ZAP-70 and 5 Lat clusters, and dashed blue curves have 20 ZAP-70 and 20 Lat clusters. Each cluster contains 20 molecules, with solid lines (dark) corresponding to the equivalent homogeneously distributed initial conditions. (**B**) The effect of increasing the initial concentration of active ZAP-70. The results are shown for 5 ZAP-70 and 5 Lat clusters. Blue curves have 100 pZAP-70 per cluster, red curves have 20 pZAP-70 per cluster, and the concentration of phosphatase is 800 per π µm^2^. **(C)** The effect of decreasing the concentration of phosphatase. Results are shown for 5 ZAP-70 and 5 Lat clusters, each of which contains 20 molecules. Red curves have 800 phosphatase per π µm^2^ and blue curves have 200 phosphatase per π µm^2^.

**Figure 4:**
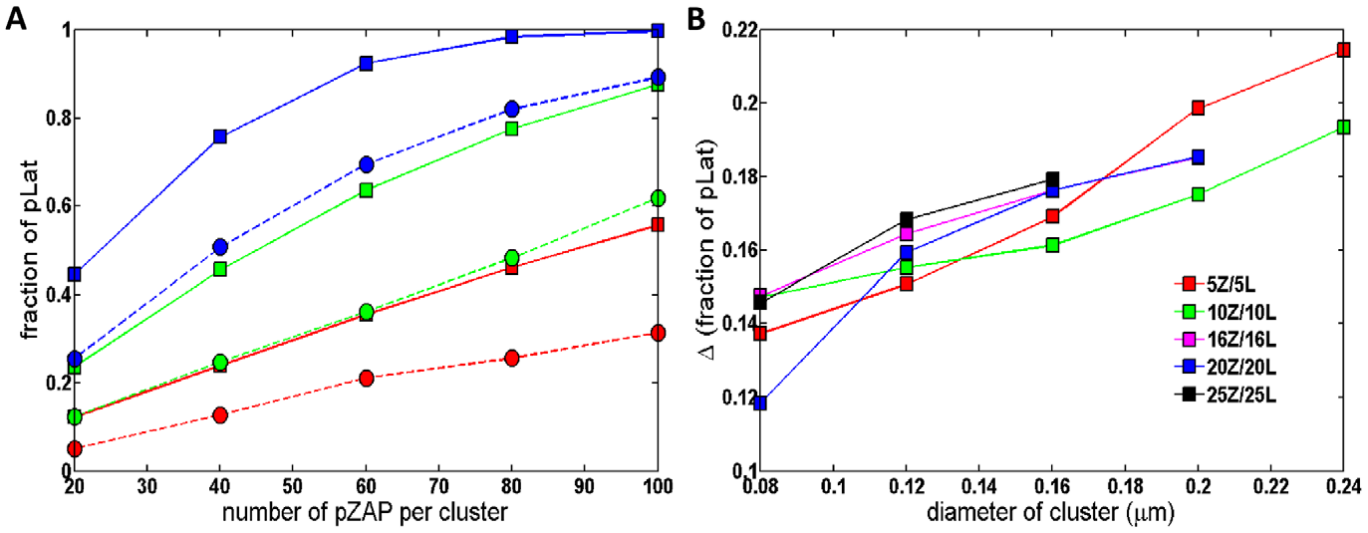
Steady-state fraction of pLat for various conditions. Each system contains 800 phosphatases of pZAP-70. We obtain the fraction of pLat over 60 different configurations of clusters with 25 trajectories for each configuration. The homogenous system produces larger quantities of pLat at all conditions. (**A**) compares the steady state fraction of pLat for various numbers of pZAP-70 per cluster. In the system with clustered proteins, 20 Lat molecules and a variable number of pZAP-70 molecules are initially located inside each cluster. Solid curves with squares and dashed curves with circles represent the homogenous systems and the cluster systems, respectively. Colors correspond to the following conditions: 20 ZAP-70 and 20 Lat clusters (blue), 10 ZAP-70 and 10 Lat clusters (green), 5 ZAP-70 and 5 Lat clusters (red). (**B**) shows the difference between the fraction of pLat in the homogenous system and the fraction of pLat in the cluster system for various diameters of clusters. Each system contains 400 Lat, and 400 pZAP-70 molecules. “5Z/5L” stands for the cluster system that contains 5 pZAP-70 and 5 Lat clusters, each of which initially contains 80 molecules.

When we include phosphatase proteins in the system, pZAP-70 molecules can be deactivated by the phosphatase. Since we start with a fixed number of pZAP-70 molecules, it is possible for all of the pZAP-70 to be deactivated before they phosphorylate all of the Lat proteins. Figure 3 compares signaling responses of systems with homogeneous and clustered proteins in the presence of phosphatase, for various numbers of clusters and various amounts of ZAP-70 per cluster. Figure 4 compares the number of pLat at steady state in the homogeneous system and the system with clustered proteins for a variety of conditions, including varied cluster size and varied number of pZAP-70 per cluster. For all conditions, the homogeneous system produces larger amounts of phosphorylated Lat on average than system with the same number of clustered proteins (see also Figures S1, S2, S3, and S4). Hence, in our simulation model, pre-existing clusters on the membrane suppress the signaling output (as measured by pLat), given a fixed amount of pZAP-70 as input. It is known that Lat dephosphorylation can occur on similar timescales to those appearing in Figs. 2 and 3 [28]. Figure S5 presents simulation results showing that explicitly considering phosphatases of Lat does not change the qualitative results: Protein clustering suppresses the magnitude of transient peaks in the number of pLat compared with homogeneously distributed proteins.

In the simulations, we have not accounted for the dynamics of pZAP-70 production, which could be influenced by the presence of TCR clusters. As discussed in the Introduction, the production of pZAP-70 begins with the ligation of TCRs by pMHC and involves multiple signaling molecules, including the kinase Lck. It is possible that clustering enhances the production of pZAP-70 by locally increasing the concentration of relevant signaling molecules. If this is the case and TCR clustering leads to larger numbers of pZAP-70 compared with an equivalent homogeneously distributed system, then the system with clustered proteins could lead to higher levels of downstream signaling. For example, in Fig. 3C, if each cluster starts with 100 pZAP-70, the system with clustered proteins results in higher amounts of pLat than the homogeneous case with 5 times fewer pZAP-70 molecules. Note that starting with 100 pZAP-70 per cluster is a physiologically unlikely number, given that TCR clusters on resting cells contain on order of 10 to 20 TCR molecules. As we discuss in the Discussion section, clustering of TCR and Lat on the cell surface may be a means for the T cell to suppress spurious triggering caused by small amounts of transiently produced ZAP-70 in the absence of antigenic peptides.

### Analysis of the Effects of the Initial Molecule Distribution

Our simulation results indicate that given a fixed amount of active ZAP-70, a system with homogeneously distributed proteins responds faster on average than a system with clustered proteins. Additionally, in the presence of phosphatases, the homogenous system produces a larger amount of phosphorylated Lat at long times, implying that the magnitude of the response is larger in the homogeneous case. By independently varying the initial concentration of active ZAP-70 and phosphatases, we consistently find that the homogenous system produces a faster response and a greater fraction of phosphorylated Lat at long times (see Figures S1, S2, and S3). In this section, we first explore features of the short-time dynamics by using scaling analysis to determine the characteristic time associated with the nearest ZAP-70 molecule diffusing into contact with a generic Lat molecule. We then compute numerically the average distance from a Lat molecule to the k^th^ nearest pZAP-70 molecule and investigate how many Lat share the same nearest neighbor. We are interested in how differences in the initial distribution of proteins lead to differences in signaling output.

We begin by considering a specific Lat molecule and estimate the characteristic distance to the nearest ZAP-70 molecule. From this we can obtain a characteristic time for the two molecules to diffuse into contact and compare the effects of the distribution of molecules on the time for the two to first meet. In the system with homogeneously distributed molecules, the ZAP-70 molecules are distributed uniformly at random, and the average distance from a Lat molecule to the nearest ZAP-70 molecule is well-approximated by.
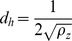
where ρ_z_ is the concentration of ZAP-70 molecules and d_h_ is given by the mean distance to the nearest neighbor distributed according to the spatial Poisson process of density ρ_z_ in continuous space. The expected time for the two molecules to diffuse this distance, with D_tot_ the sum of the individual diffusion coefficients, is



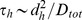
In the system with clustered proteins, the concentration of ZAP-70 clusters is ρ_c_ =  ρ_z_/n_z_, where n_z_ denotes the number of ZAP-70 molecules per cluster. Hence, the expected distance from the edge of a Lat cluster to the edge of a ZAP-70 cluster is



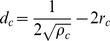
where r_c_ denotes the radius of a cluster. Thus, the expected time for a ZAP-70 molecule to diffuse into contact with a Lat molecule scales as τ_c_ ∼ d_c_^2^/D, plus corrections due to the molecules not being at the edge of the cluster on average (these corrections are typically small compared with τ_c_). Note that the diffusion coefficient used here is solely that of the ZAP-70 molecule, since Lat remains confined in its cluster. By comparing τ_c_ and τ_h_, we find that the homogeneous system is characterized by a shorter expected time for the first encounter when the number of clusters is sufficiently small. For example, with 5 TCR and 5 Lat clusters, each containing 20 molecules, the expected first encounter time is an order of magnitude faster in the homogeneously distributed system. As the number of clusters increases, the difference between τ_c_ with τ_h_ becomes smaller, suggesting the need for more detailed analysis and leading us to address the distribution of distances from a Lat molecule to ZAP-70 molecules numerically.

**Figure 5:**
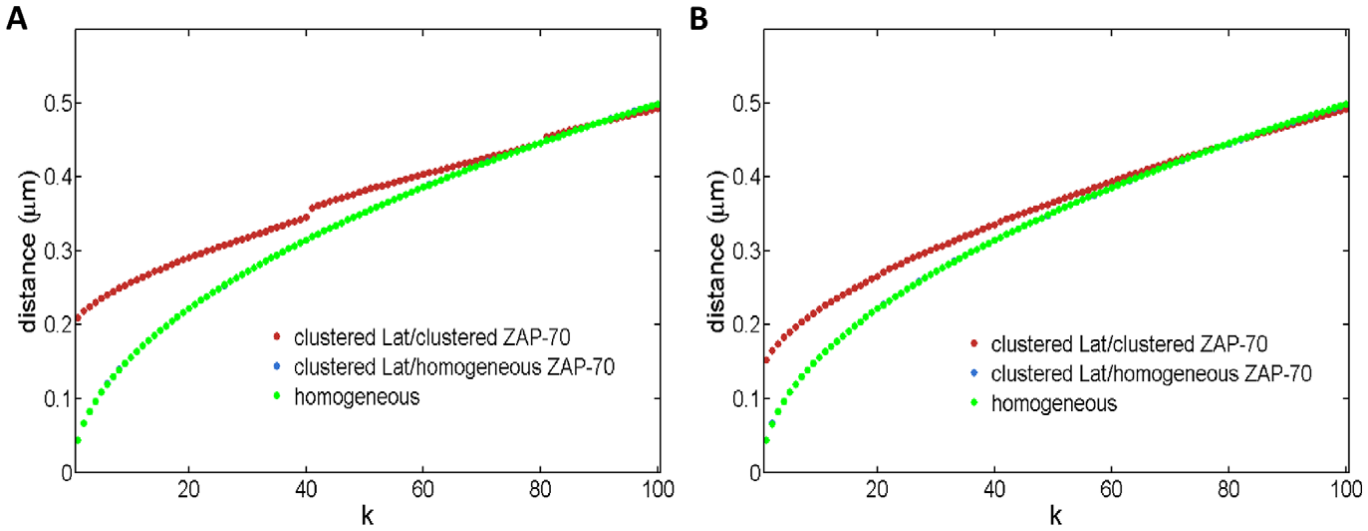
The mean distance from a Lat molecule to the k^th^ nearest pZAP-70. Results are averaged over 1000 initial configurations. Note that in both figures, blue and green data points are nearly indistinguishable. (**A**) Results for 10 Lat clusters, each of which contains 40 molecules (400 Lat molecules are distributed homogeneously in the green results). There are 400 pZAP-70 molecules, either homogeneously distributed or distributed into 10 clusters. (**B**) Results for 20 Lat clusters, each of which contains 20 molecules. There are 400 pZAP-70 molecules, either homogeneously distributed or distributed into 20 clusters. To avoid edge effects, numerical results were obtained by considering only Lat molecules near the center of the system.

We begin by computing the average distance from a Lat molecule to the k^th^ nearest ZAP-70 molecule for various initial conditions. Figures 5 and S6 demonstrate that reactants are farther apart on average when all molecules are confined to clusters compared with cases in which at least one of the species is homogeneously distributed. As suggested above, it follows that the average time for reactants to first diffuse into contact is larger in the system in which proteins are clustered. In Fig. S8, we present simulation results for a typical system and plot the cumulative probability, as a function of time, that a single pZAP-70 molecule has bound a Lat molecule.

**Figure 6:**
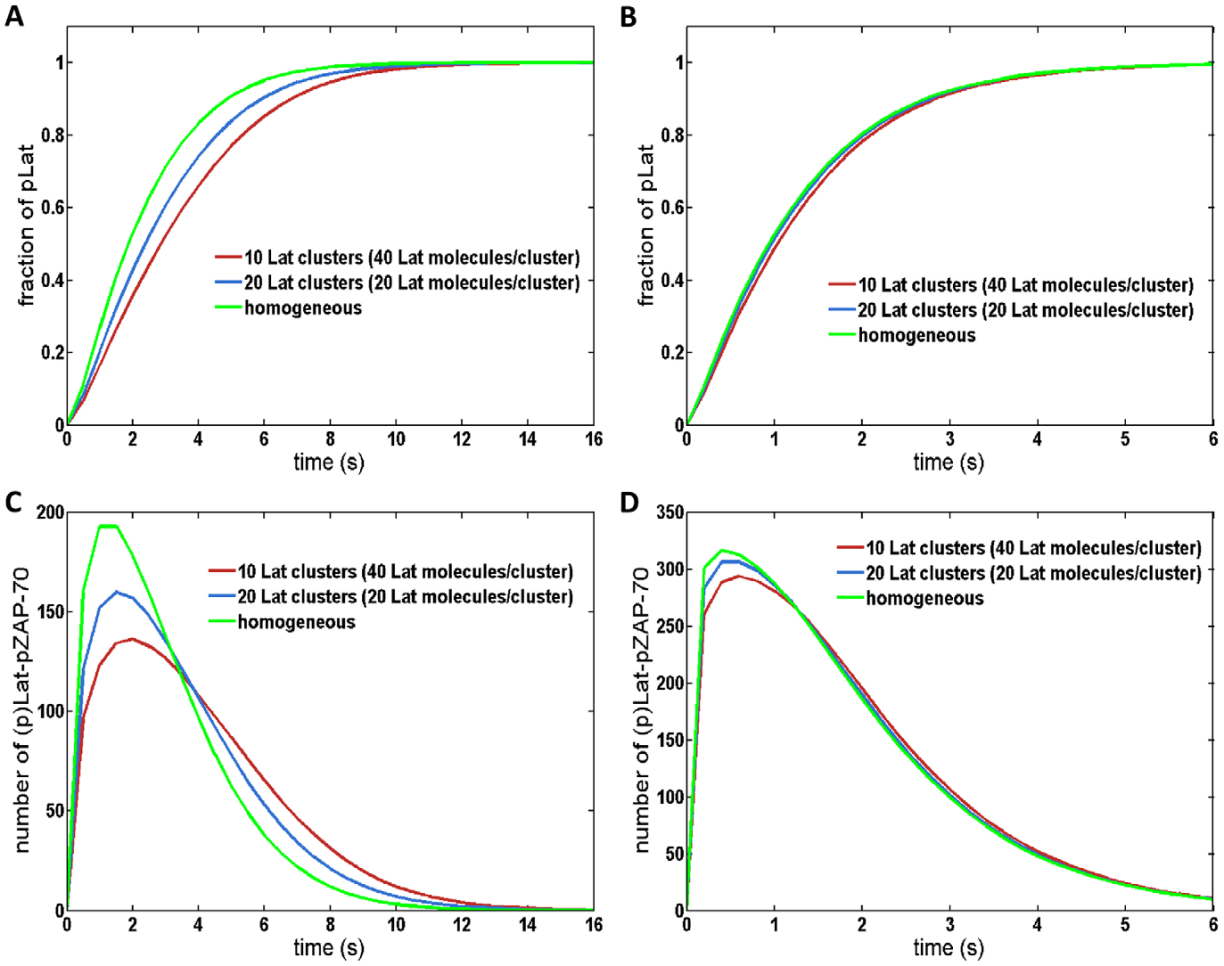
The effect of distributing a fixed number of Lat molecules in different numbers of clusters. Simulation results for 400 Lat molecules placed in 10 clusters (red), 20 clusters (blue), or homogeneously (geen). In all cases, 400 pZAP-70 molecules are homogeneously distributed initially and can diffuse through all domains. No phosphatase is present. (**A**) and (**B**) display the fraction of pLat as a function of time for D = 0.0033 µm^2^/s and D = 0.33 µm^2^/s, respectively. (**C**) and (**D**) measure the number of ZAP-70 bound to Lat as a function of time and correspond to the diffusivities of (A) and (B), respectively. The differences between the different initial configurations are more pronounced at slower diffusion rates.

**Figure 7:**
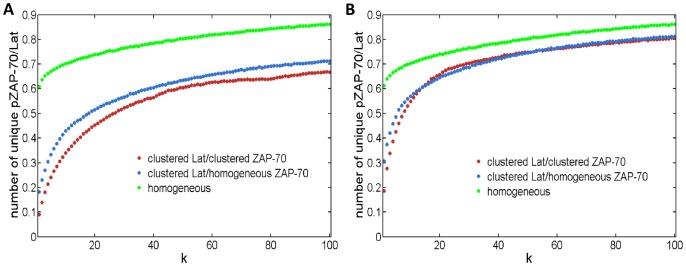
The number of unique neighboring pZAP-70 per Lat molecule. Results are averaged over 1000 initial configurations. (**A**) Results for 10 Lat clusters, each of which contains 40 molecules (400 Lat molecules are homogeneously distributed in the green results). There are 400 pZAP-70 molecules, either homogeneously distributed or distributed into 10 clusters. (**B**) Results for 20 Lat clusters, each of which contains 20 molecules. There are 400 pZAP-70 molecules, either homogeneously distributed or distributed into 20 clusters.

As can be seen from results in Figs. 3 and 4, for a fixed number of phosphatases, the magnitude of the signaling response is smaller in the system with clustered proteins than in the homogeneous system. In order to phosphorylate a Lat molecule, an active ZAP-70 must not be dephosphorylated by a phosphatase before it encounters the Lat molecule. Since the phosphatases are uniformly distributed, the longer a ZAP-70 molecule takes to diffuse into contact with a Lat molecule, the more likely it is to be inactivated. To first approximation, the probability that a ZAP-70 molecule is active when encountering a Lat molecule is (1-p_deact_)^λ^, where p_deact_ is the probability that a phosphatase deactivates a ZAP-70 molecule when they occupy the same lattice site and λ denotes the expected number of encounters between the ZAP-70 and a phosphatase before the ZAP-70 encounters a Lat molecule. Assuming the phosphatases are well mixed, λ is expected to increase linearly with time. Hence, there is a smaller probability for a ZAP-70 molecule to be active when it first encounters a Lat molecule in the case with clustered proteins since the expected time for such an encounter is longer than in the homogeneous case.

We further investigated how the initial distribution of proteins affects the overall signaling response when ZAP-70 molecules are homogenously distributed but Lat is clustered. In this case, the distance from a Lat molecule to the k^th^ nearest ZAP-70 is the same as in the homogenous system (see Fig. 5). However, at low concentrations of pZAP-70 or in systems with small clusters, few pZAP-70 are initially found within the clusters, and the time to diffuse into a cluster increases the first encounter time relative to the homogeneous case since the time for ZAP-70 to diffuse into a cluster depends only on the ZAP-70 diffusion coefficient. At sufficiently high pZAP-70 concentrations, such as those used to obtain the results in Fig. 6, the characteristic time to a first encounter is expected to occur on a comparable time scale to the homogenous system. Even with a comparable time scale, we find that the overall response of this system is slower than the homogeneous system. To illustrate another physical feature that accounts for differences in the signaling response, we find the average number of unique neighboring ZAP-70 per Lat molecule. For a given initial configuration, we find the nearest ZAP-70 molecule to every Lat molecule and determine the total number of unique ZAP-70 molecules represented. This gives the number of unique neighboring ZAP-70 per Lat molecule, which provides a measure of how many Lat share the same ZAP-70 as a nearest neighbor. If the number is small, many Lat share the same nearest neighbor ZAP-70. Hence, the expected first encounter time is not representative of the characteristic time for most Lat to first encounter a ZAP-70, since the first Lat to bind the neighboring ZAP-70 will effectively sequester it and ZAP-70 from greater distances must diffuse into contact with the other Lat. In Fig. 7, we present the average number of unique neighboring ZAP-70 per Lat molecule, showing that it is highest in the homogeneous case. Additionally, in Fig. 6, it is seen that increasing the number of Lat clusters while keeping the total number of Lat and ZAP-70 fixed leads to a faster response time. Increasing the number of clusters does not change the average closest distance from a Lat molecule to a ZAP-70 molecule, but it increases the average number of distinct nearest ZAP-70 molecules per Lat molecule, as can be seen by comparing Figs. 7A and 7B. This implies that, at early times, increasing the number of clusters increases the number of encounters per unit time by increasing the number of distinct nearest ZAP-70 molecules per Lat. As is seen in Fig. 6, increasing the number of Lat clusters with fixed number of molecules increases the number of pZAP-70 bound to Lat and increases phosphorylated Lat production at early times. We also considered how the changes of other parameters affect the production of pLat (see Figures S9, S10, and S11). For example, reducing the size of clusters decreases the number of unique neighboring ZAP-70 per Lat and increases the diffusion time for pZAP-70 to enter Lat clusters, thus reducing the speed of the signaling response (see Figures S4, S7, and S11).

## Discussion

In this paper, we investigated effects of protein clustering at the cell membrane on early signaling events in T cell activation against antigen. This was accomplished by comparing the output of a model system in which the initial distribution of proteins was either homogeneous or clustered. We focused on the phosphorylation of Lat by active ZAP-70 molecules that originate from TCR clusters, using stochastic computer simulations to study the dynamics of Lat phosphorylation. Our main finding is that the clustering of Lat and TCR suppresses signaling when active ZAP-70 must diffuse into contact with Lat.

We examined two different systems with clustered proteins, comparing their signaling response to the homogeneously distributed system. In the first, active pZAP-70 molecules originate from TCR clusters and diffuse on the membrane in search of clustered Lat molecules. We found that the characteristic time for the initial encounters between pZAP-70 and Lat is greater than in the homogenous system. Since a pZAP-70 takes longer to find Lat, in the presence of a phosphatase of ZAP-70, the chance for a pZAP-70 to phosphorylate Lat molecules is smaller in the system with clustered proteins. This is because it is more likely that active ZAP-70 will be dephosphorylated before encountering Lat when proteins are clustered. In the second inhomogeneous system, Lat is confined to clusters, but pZAP-70 is initially distributed homogeneously. From analysis of this system, it was found that reducing the number of Lat clusters with a fixed total number of Lat molecules resulted in slower production of phosphorylated Lat, even though the nearest neighbor distribution is identical. This highlights that the number of Lat molecules sharing the same nearest ZAP-70 molecule also plays a role in the signaling response. Overall, in both systems we have found that the homogeneous system reaches steady state faster and yields a larger signaling output (as measured by phosphorylated Lat), indicating that the clustering of proteins suppresses the phosphorylation of Lat molecules on the membrane.

Our results suggest that the clustering of proteins on the cell membrane may provide a regulatory mechanism by which T cells suppress transient, spurious signals initiated by stimulation of TCRs by endogenous peptides. Such stimulation is likely to produce small numbers of activated ZAP-70, and by spatially segregating TCR and Lat clusters, the T cell can minimize the effects of spurious ZAP-70 activation. However, the separation of clusters may come at the expense of sensitivity to antigenic peptides. Recent experimental results suggest novel signaling mechanisms that may give insight into how T cells produce large amounts of downstream signal even though protein clustering on the membrane seems to suppress such production [11], [17], [25], [29]. Two studies indicate that TCR and Lat clusters are brought into close proximity after the T cell first recognizes antigenic peptides: The results of Lillemeier et al. reveal that clusters of TCR and Lat molecules on the membrane appear to come closer together upon stimulation by antigen [11], while the recent findings of Williamson et al. suggest that most surface-bound Lat do not participate in T cell activation and that most Lat phosphorylated during T cell activation reside on intracellular, membrane-recruited vesicles [25], [30]. In the first mechanism, clusters brought into close proximity would result in ZAP-70 having to diffuse shorter distances to phosphorylate Lat. Additionally, the proximity could facilitate direct interaction in which ZAP-70 bound to TCR complexes near the edge of T cell clusters could directly phosphorylate Lat molecules. In the second mechanism, the recruitment of vesicles containing Lat molecules to the membrane could result in direct contact between TCR clusters and Lat-containing vesicles, yielding a highly concentrated local environment in which interactions between ZAP-70 and Lat are very likely. Since membrane-bound Lat are apparently not used to initiate downstream signaling in this model, it is likely beneficial for the resting T cell to keep them organized into clusters, since this decreases the chance of spurious activation by transiently-bound endogenous peptides. A third mechanism was proposed by Sherman et al., whose recent work suggests that TCR and Lat clusters partially overlap, leading the authors to propose that Lat is phosphorylated by ZAP-70 in or near the overlapping regions [17]. In each of the proposed mechanisms, it is possible that ZAP-70 can be transiently activated and unbind from the cytoplasmic region of the TCR complex. Having molecules clustered may help to suppress spurious downstream signaling, while the mechanisms above may provide a means for cells to amplify signals when antigenic peptides are encountered by TCR.

It remains to explore other ways that protein clusters may influence T cell activation. As discussed previously, clustering of TCR on the cell membrane may enhance the production of pZAP-70 in response to antigenic peptides as a result of the enhancement of Lck activity within the cluster. The clustering of Lat may also enhance downstream signaling events by increasing the local concentration of various signaling proteins. One example is the protein SOS, which is recruited by phosphorylated Lat and activates the important signaling protein Ras by means of a Ras-influenced positive feedback loop. But, the early signaling event of Lat phosphorylation by ZAP-70 seems to be suppressed by the clustering of proteins on the T cell surface.

## Supporting Information

Figure S1 **The effect of concentration of pZAP-70 on the production of pLat.** Each cluster system contains 5 ZAP-70 and 5 Lat clusters. There is no phosphatase. “20 pZAP-70, c” stands for the cluster system in which each cluster initially contains 20 pZAP-70. “20 pZAP-70, h” is the homogenous system that contains the same amount of reactants as “20 pZAP-70, c.” Each Lat cluster contains 20 Lat molecules. See Figure 2 for a description of simulation method.
(TIF)

Figure S2 **The effect of the presence of phosphatase of pZAP-70 at various concentrations of pZAP-70 on the production of pLat.** Each system contains 800 phosphatases per π µm^2^. In the system with clustered proteins, ZAP-70 molecules are initially located inside their clusters. See Figure S1 for a description of legend terminology.
(TIF)

Figure S3 **The effect of phosphatase concentration on the production of pLat.** Each cluster contains 20 molecules. “200 P, h” and “200 P, c” stand for the homogenous system and the cluster system, respectively, that contains 200 phosphatases. In the system with clustered proteins, ZAP-70 molecules are initially located inside their clusters. Each cluster system contains 5 ZAP-70 and 5 Lat clusters and each cluster contains 20 molecules.
(TIF)

Figure S4 **Fraction of pLat in the cluster system for various cluster sizes in the presence of no phosphatases.** Each system contains 400 Lat and 400 pZAP-70 molecules. “5Z/5L, c (0.08 µm)” denotes the cluster system that consists of clusters with a diameter of 0.08 µm, while “5Z/5L, h” denotes the homogeneous system. We obtain the fraction of pLat over 60 different cluster-system configurations with 25 trajectories for each configuration. **(A)** pZAP-70 molecules are initially confined inside clusters. **(B)** pZAP-70 molecules are initially homogenously distributed.
(TIF)

Figure S5 **Fraction of pLat as a function of time, with dephosphorylation of Lat molecules.** Each system contains 800 phosphatases of pZAP-70. In the system with clustered proteins, each ZAP-70 and Lat cluster initially contains 20 molecules. We obtain the average fraction of pLat over 20 different cluster-system configurations with 25 trajectories for each configuration. “5Z/5L, c” stands for the cluster system that contains 5 pZAP and 5 Lat clusters. “5Z/5L, h” is the homogeneous system that contains the same number of reactants as in the “5Z/5L, c” system. **(A)** 800 phosphatases of pLat **(B)** 80 phosphatases of pLat
(TIF)

Figure S6 **The effect of reduced cluster number on the mean distance from a Lat molecule to the k^th^ nearest pZAP-70 and the number of unique pZAP-70 per Lat.** The “clustered Lat/cluster ZAP-70” system contains 5 Lat and 5 ZAP-70 clusters, each of which contains 20 molecules. We obtain the mean distances and the number of unique pZAP-70 per Lat by averaging over 1000 configurations. See Figure 5 for a description of legend terminology.
(TIF)

Figure S7 **The effect of reduced size of clusters on the mean distance from a Lat molecule to the k^th^ nearest pZAP-70 and the number of unique pZAP-70 per Lat.** The “clustered Lat/cluster ZAP-70” system contains 20 Lat and 20 ZAP-70 clusters. The diameter of each cluster is reduced by a factor of 2. We obtain the mean distances and the number of unique pZAP-70 per Lat by averaging over 1000 configurations.
(TIF)

Figure S8 **The cumulative probability of observing Lat-pZAP binding as a function of time for a single pZAP.** Each system contains 400 Lat molecules, either homogeneously distributed or confined in clusters. One pZAP-70 is randomly located initially, and we determine the probability that the first binding between the pZAP-70 and a Lat molecules has occurred by a given time. For the cluster system, we obtain the probability of observing Lat-pZAP-70 over 100 different configurations with 50 trajectories for each configuration. The diffusivity of molecules in system is 0.0033 µm^2^/s.
(TIF)

Figure S9 **The effect of a change in the rate constant for the association or dissociation between pZAP and Lat.** Other kinetic parameters are the same as those in Table S1. See Figure 6 for a description of legend terminology. **(A)** The rate constant *K_on,pZAP_*_70*−Lat*_ is reduced by a factor of 2000. **(B)** The rate constant *K_off,pZAP_*_70*−Lat*_ is increased by a factor of 2000.
(TIF)

Figure S10 **The effect of a change in the amount of reactants.** Other parameters are the same as those in Table S1 and Table S2. **(A)** The number of pZAP-70 in the system is reduced by a factor of 10. **(B)** The number of Lat in the system is reduced by a factor of 10.
(TIF)

Figure S11 **The effect of a decrease in the size of each cluster.** The diameter of each cluster is reduced by a factor of 2.
(TIF)

Table S1 Rate constants for simulations.
(DOCX)

Table S2 Concentration of species for simulations.
(DOCX)

Materials and Methods (DOCX)
