# Correction: Protein Clusters on the T Cell Surface May Suppress Spurious Early Signaling Events

**DOI:** 10.1371/annotation/ed8e10ba-0a44-414e-8451-08b26930bfc4

**Published:** 2012-09-17

**Authors:** Woo Chung, Steven M. Abel, Arup K. Chakraborty

There were errors in the author affiliations. The correct affiliations are:

Woo Chung^1^, Steven M. Abel^1^, Arup K. Chakraborty^1,2,3,4^*

1 Department of Chemical Engineering, Massachusetts Institute of Technology, Cambridge, Massachusetts, United States of America,

2 Department of Chemistry, Massachusetts Institute of Technology, Cambridge, Massachusetts, United States of America,

3 Department of Biological Engineering, Massachusetts Institute of Technology, Cambridge, Massachusetts, United States of America,

4 Ragon Institute of MGH, MIT, and Harvard, Charlestown, Massachusetts, United States of America 

