# Supplementary figures and images for: Protein Clusters on the T Cell Surface May Suppress Spurious Early Signaling Events
Source: PLoS One. 2012 Sep 4;7(9):e44444. doi: 10.1371/journal.pone.0044444 (PMC3433417; doi:10.1371/journal.pone.0044444)

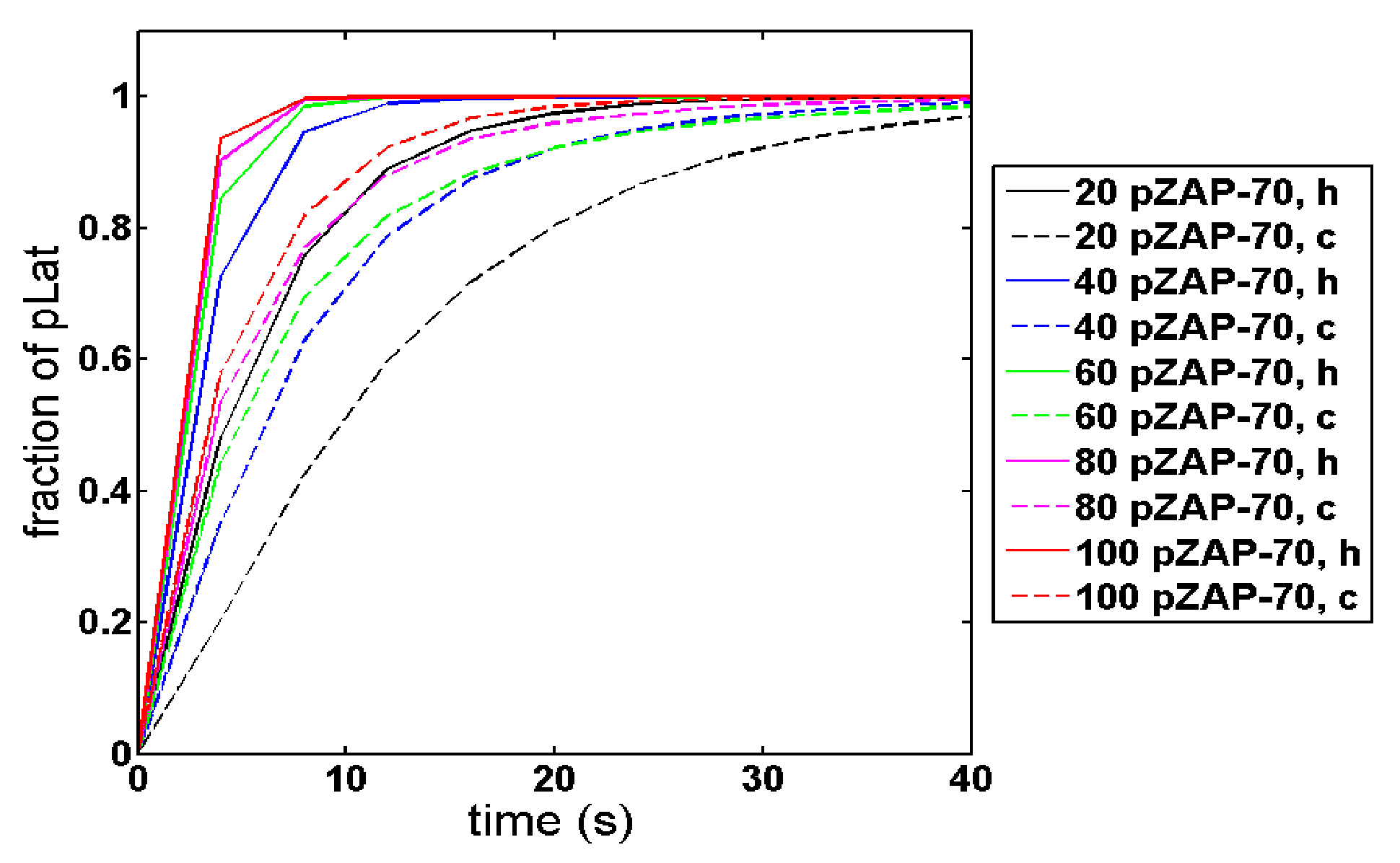

Supplement: Figure S1 — The effect of concentration of pZAP-70 on the production of pLat. Each cluster system contains 5 ZAP-70 and 5 Lat clusters. There is no phosphatase. “20 pZAP-70, c” stands for the cluster system in which each cluster initially contains 20 pZAP-70. “20 pZAP-70, h” is the homogenous system that contains the same amount of reactants as “20 pZAP-70, c.” Each Lat cluster contains 20 Lat molecules. See Figure 2 for a description of simulation method. (TIF) [file pone.0044444.s001.tif]

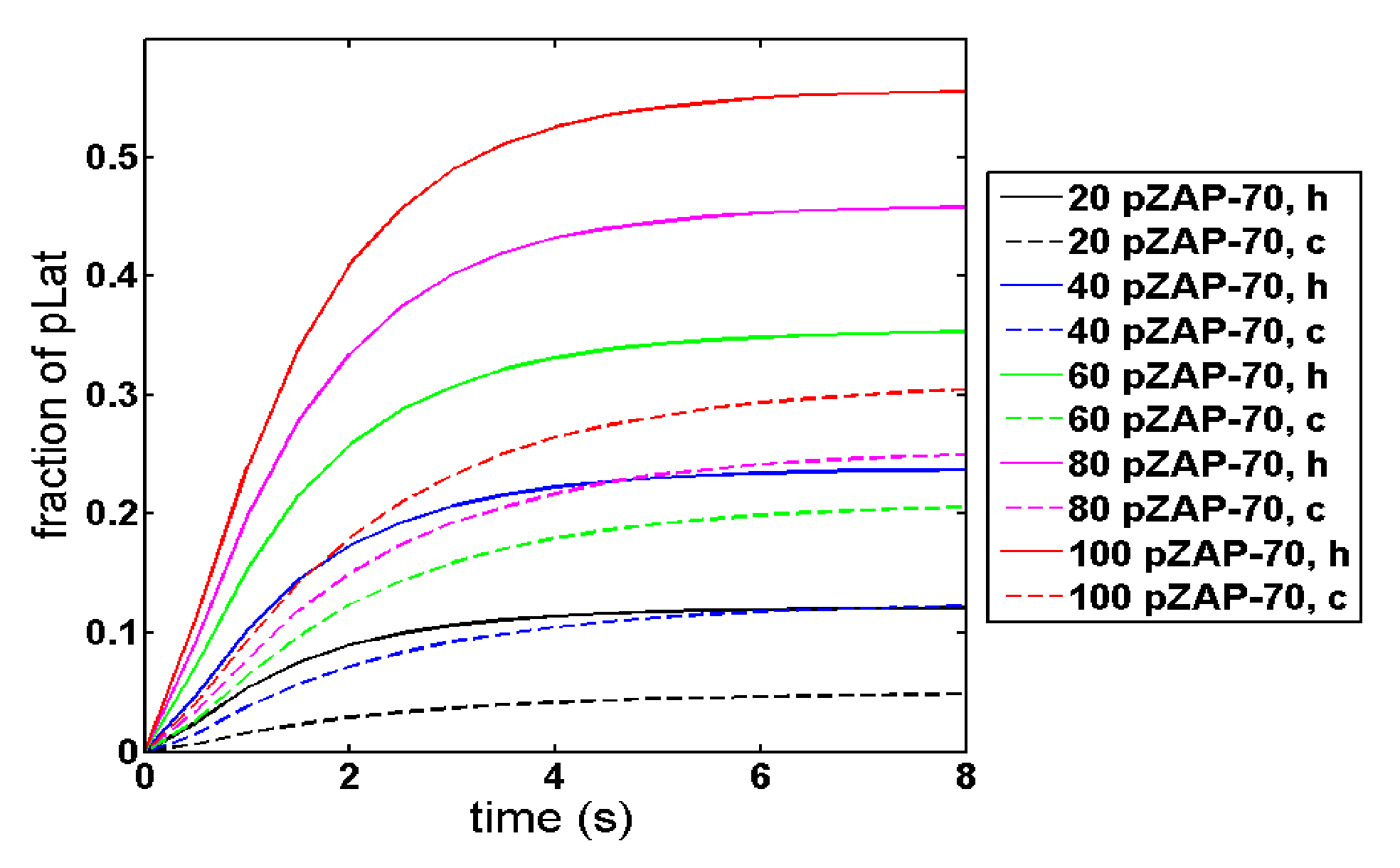

Supplement: Figure S2 — The effect of the presence of phosphatase of pZAP-70 at various concentrations of pZAP-70 on the production of pLat. Each system contains 800 phosphatases per π µm2. In the system with clustered proteins, ZAP-70 molecules are initially located inside their clusters. See Figure S1 for a description of legend terminology. (TIF) [file pone.0044444.s002.tif]

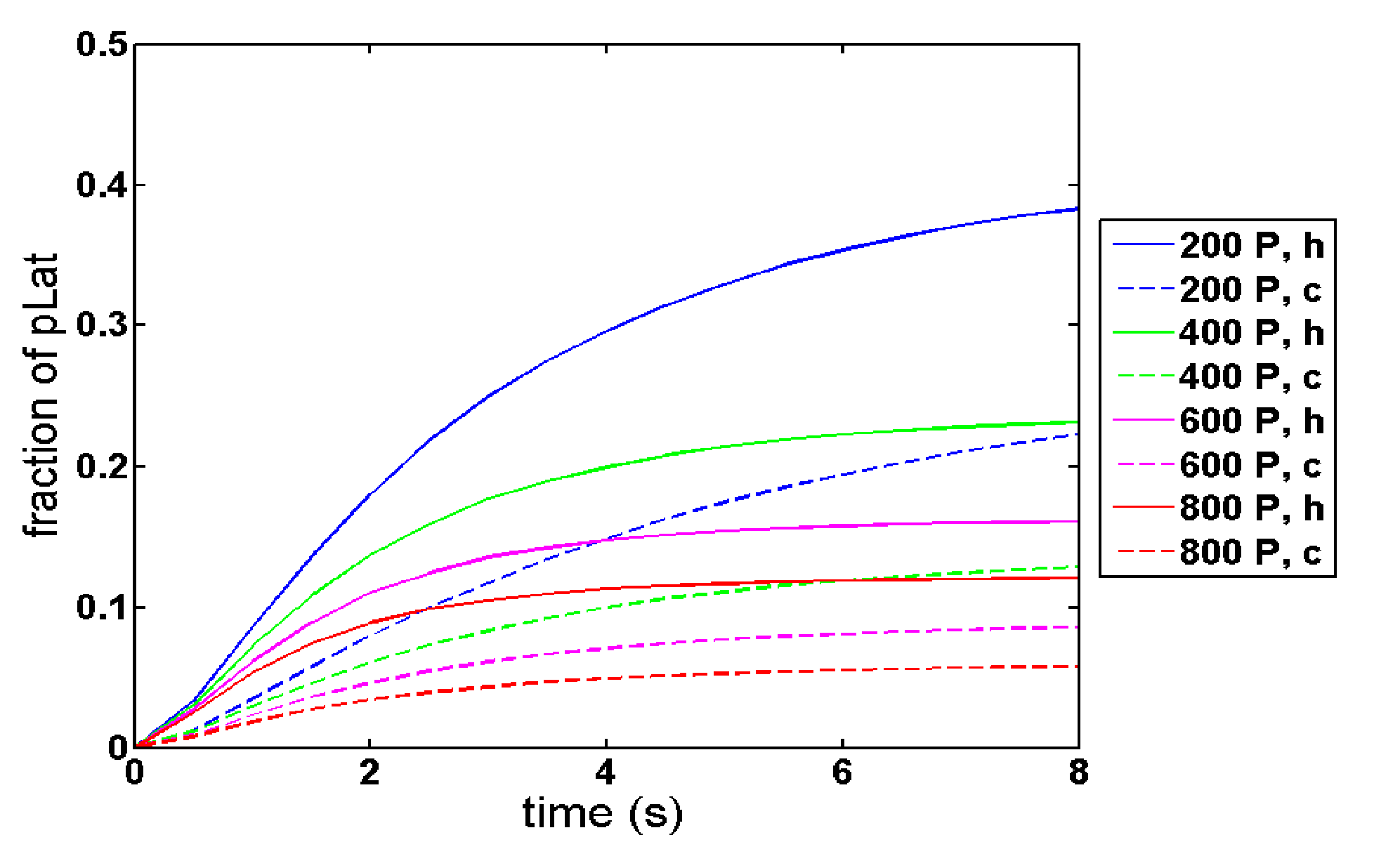

Supplement: Figure S3 — The effect of phosphatase concentration on the production of pLat. Each cluster contains 20 molecules. “200 P, h” and “200 P, c” stand for the homogenous system and the cluster system, respectively, that contains 200 phosphatases. In the system with clustered proteins, ZAP-70 molecules are initially located inside their clusters. Each cluster system contains 5 ZAP-70 and 5 Lat clusters and each cluster contains 20 molecules. (TIF) [file pone.0044444.s003.tif]

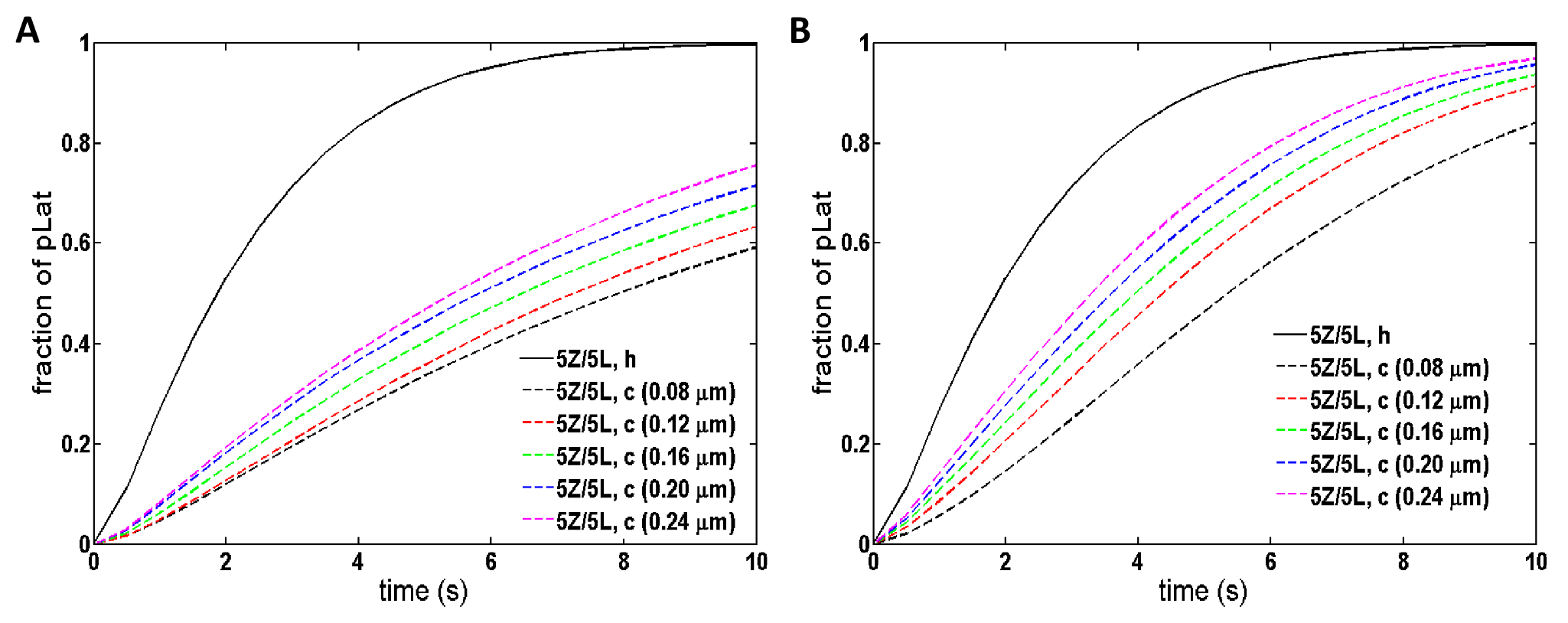

Supplement: Figure S4 — Fraction of pLat in the cluster system for various cluster sizes in the presence of no phosphatases. Each system contains 400 Lat and 400 pZAP-70 molecules. “5Z/5L, c (0.08 µm)” denotes the cluster system that consists of clusters with a diameter of 0.08 µm, while “5Z/5L, h” denotes the homogeneous system. We obtain the fraction of pLat over 60 different cluster-system configurations with 25 trajectories for each configuration. (A) pZAP-70 molecules are initially confined inside clusters. (B) pZAP-70 molecules are initially homogenously distributed. (TIF) [file pone.0044444.s004.tif]

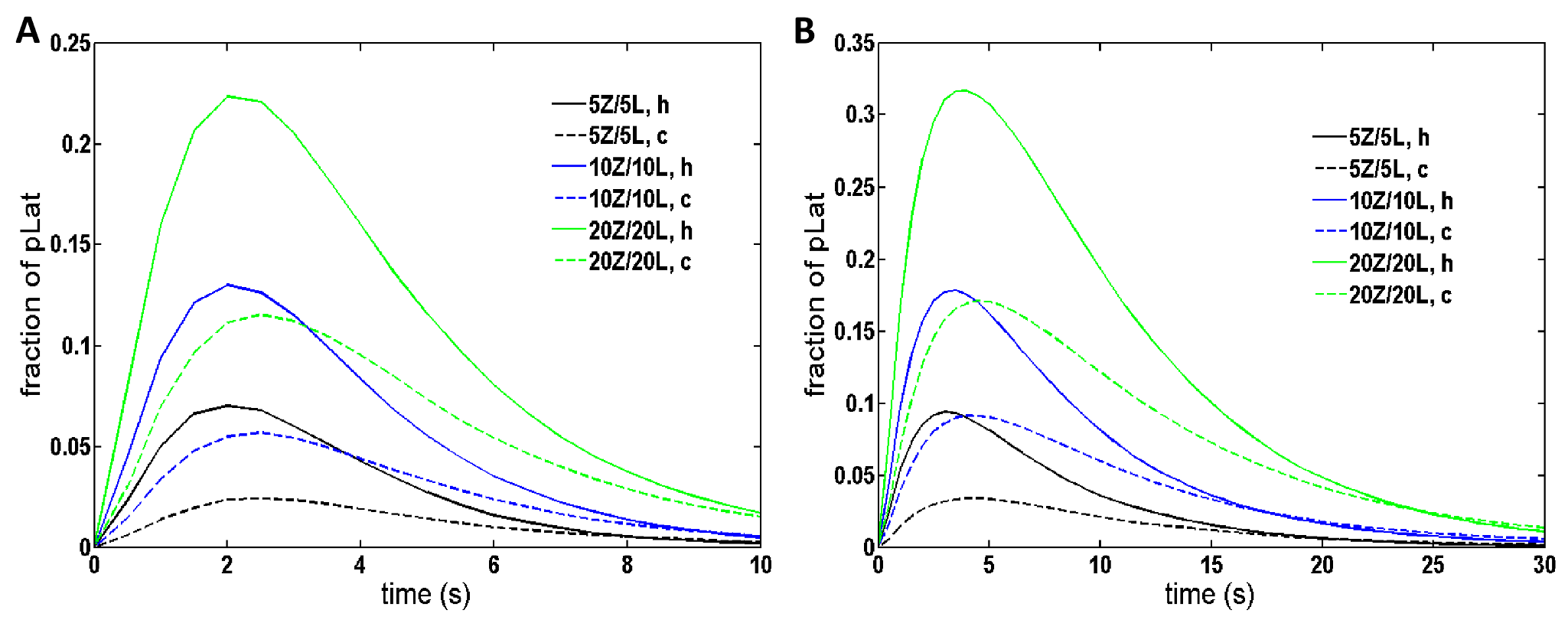

Supplement: Figure S5 — Fraction of pLat as a function of time, with dephosphorylation of Lat molecules. Each system contains 800 phosphatases of pZAP-70. In the system with clustered proteins, each ZAP-70 and Lat cluster initially contains 20 molecules. We obtain the average fraction of pLat over 20 different cluster-system configurations with 25 trajectories for each configuration. “5Z/5L, c” stands for the cluster system that contains 5 pZAP and 5 Lat clusters. “5Z/5L, h” is the homogeneous system that contains the same number of reactants as in the “5Z/5L, c” system. (A) 800 phosphatases of pLat (B) 80 phosphatases of pLat (TIF) [file pone.0044444.s005.tif]

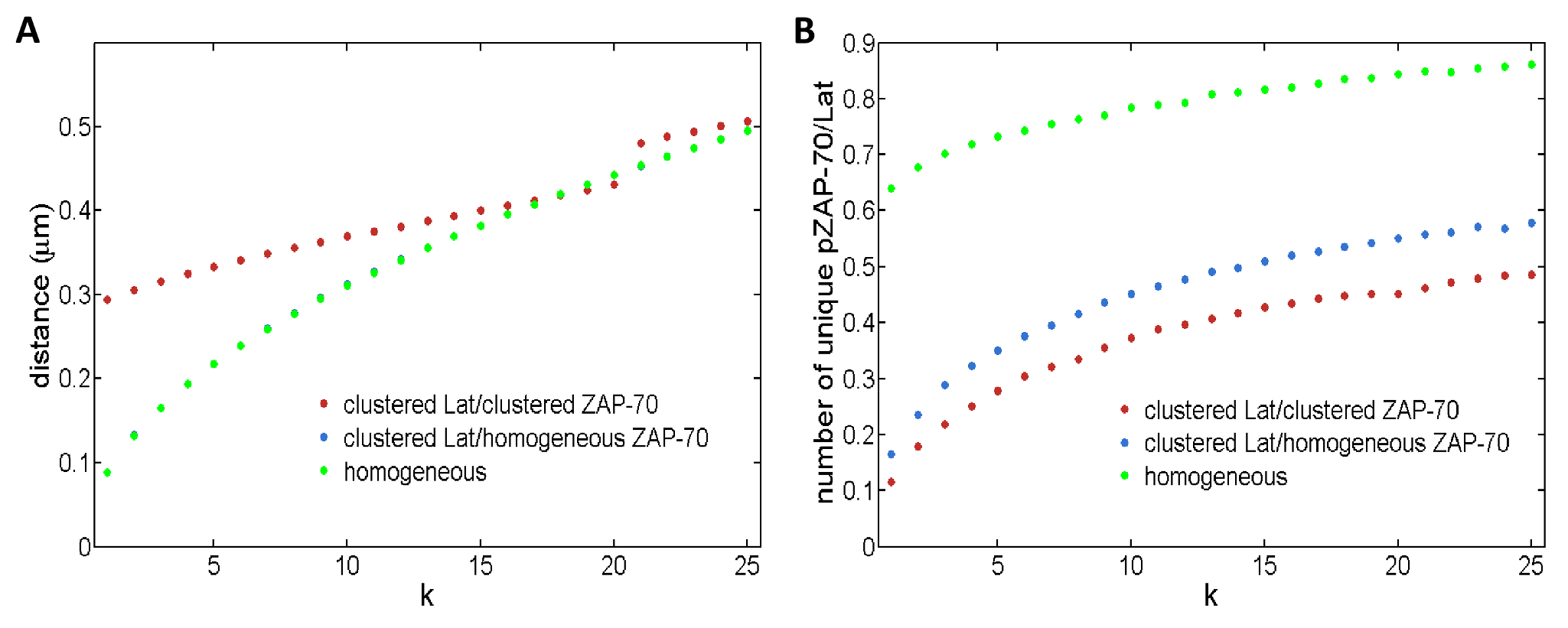

Supplement: Figure S6 — The effect of reduced cluster number on the mean distance from a Lat molecule to the kth nearest pZAP-70 and the number of unique pZAP-70 per Lat. The “clustered Lat/cluster ZAP-70” system contains 5 Lat and 5 ZAP-70 clusters, each of which contains 20 molecules. We obtain the mean distances and the number of unique pZAP-70 per Lat by averaging over 1000 configurations. See Figure 5 for a description of legend terminology. (TIF) [file pone.0044444.s006.tif]

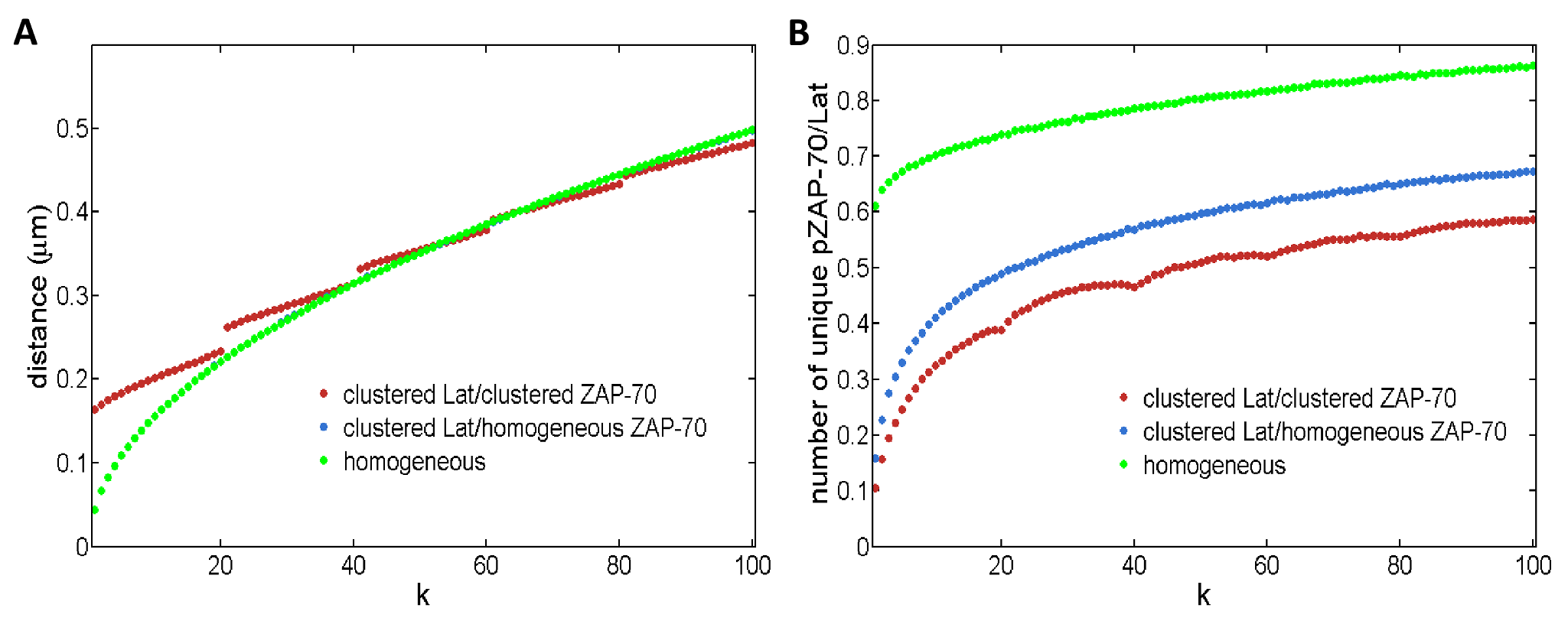

Supplement: Figure S7 — The effect of reduced size of clusters on the mean distance from a Lat molecule to the kth nearest pZAP-70 and the number of unique pZAP-70 per Lat. The “clustered Lat/cluster ZAP-70” system contains 20 Lat and 20 ZAP-70 clusters. The diameter of each cluster is reduced by a factor of 2. We obtain the mean distances and the number of unique pZAP-70 per Lat by averaging over 1000 configurations. (TIF) [file pone.0044444.s007.tif]

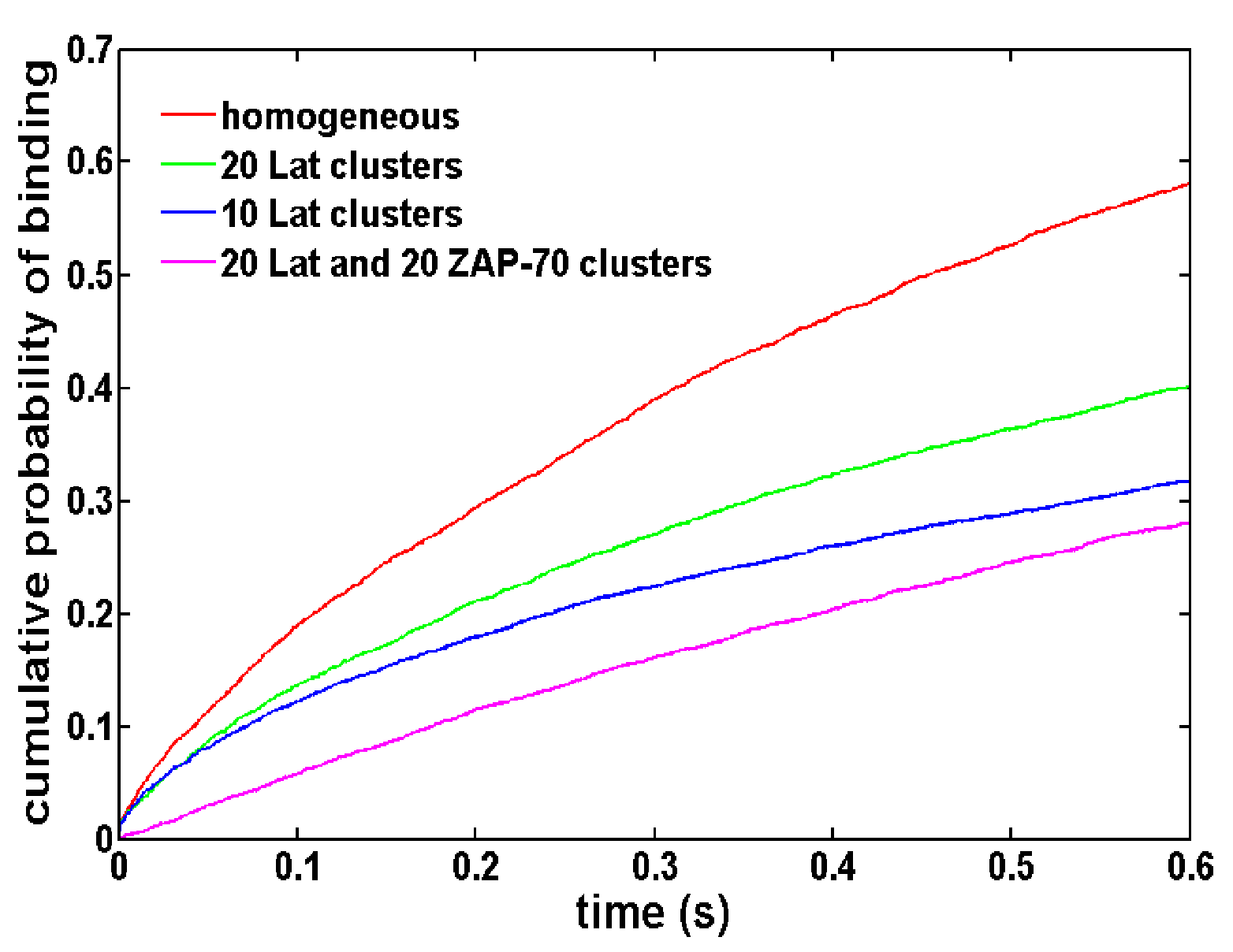

Supplement: Figure S8 — The cumulative probability of observing Lat-pZAP binding as a function of time for a single pZAP. Each system contains 400 Lat molecules, either homogeneously distributed or confined in clusters. One pZAP-70 is randomly located initially, and we determine the probability that the first binding between the pZAP-70 and a Lat molecules has occurred by a given time. For the cluster system, we obtain the probability of observing Lat-pZAP-70 over 100 different configurations with 50 trajectories for each configuration. The diffusivity of molecules in system is 0.0033 µm2/s. (TIF) [file pone.0044444.s008.tif]

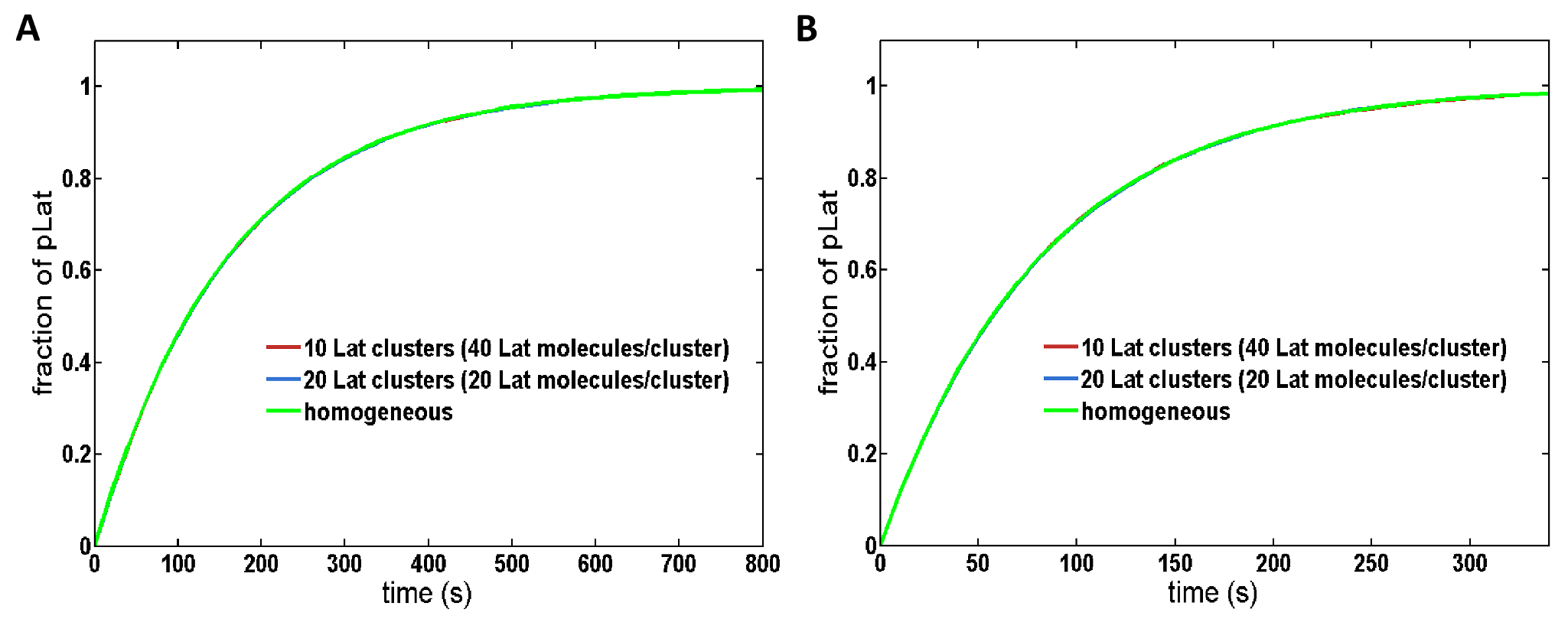

Supplement: Figure S9 — The effect of a change in the rate constant for the association or dissociation between pZAP and Lat. Other kinetic parameters are the same as those in Table S1. See Figure 6 for a description of legend terminology. (A) The rate constant Kon,pZAP 70−Lat is reduced by a factor of 2000. (B) The rate constant Koff,pZAP 70−Lat is increased by a factor of 2000. (TIF) [file pone.0044444.s009.tif]

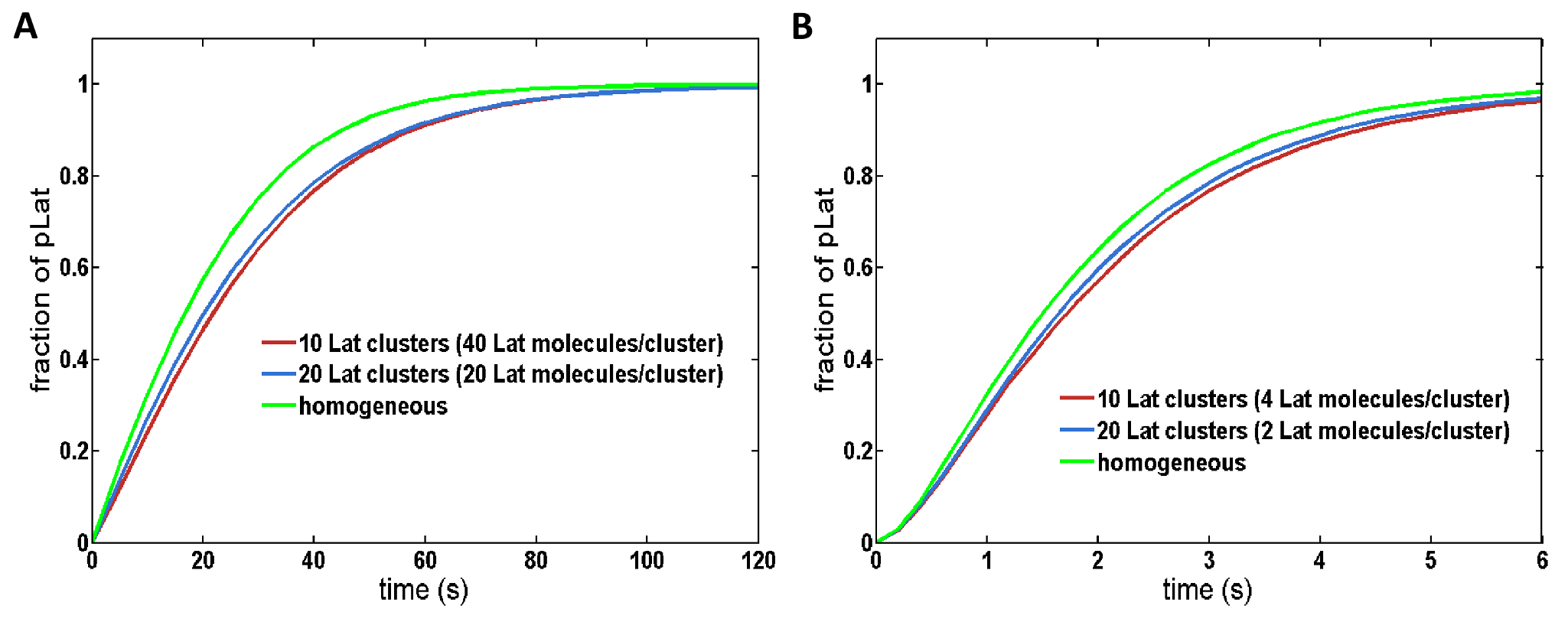

Supplement: Figure S10 — The effect of a change in the amount of reactants. Other parameters are the same as those in Table S1 and Table S2. (A) The number of pZAP-70 in the system is reduced by a factor of 10. (B) The number of Lat in the system is reduced by a factor of 10. (TIF) [file pone.0044444.s010.tif]

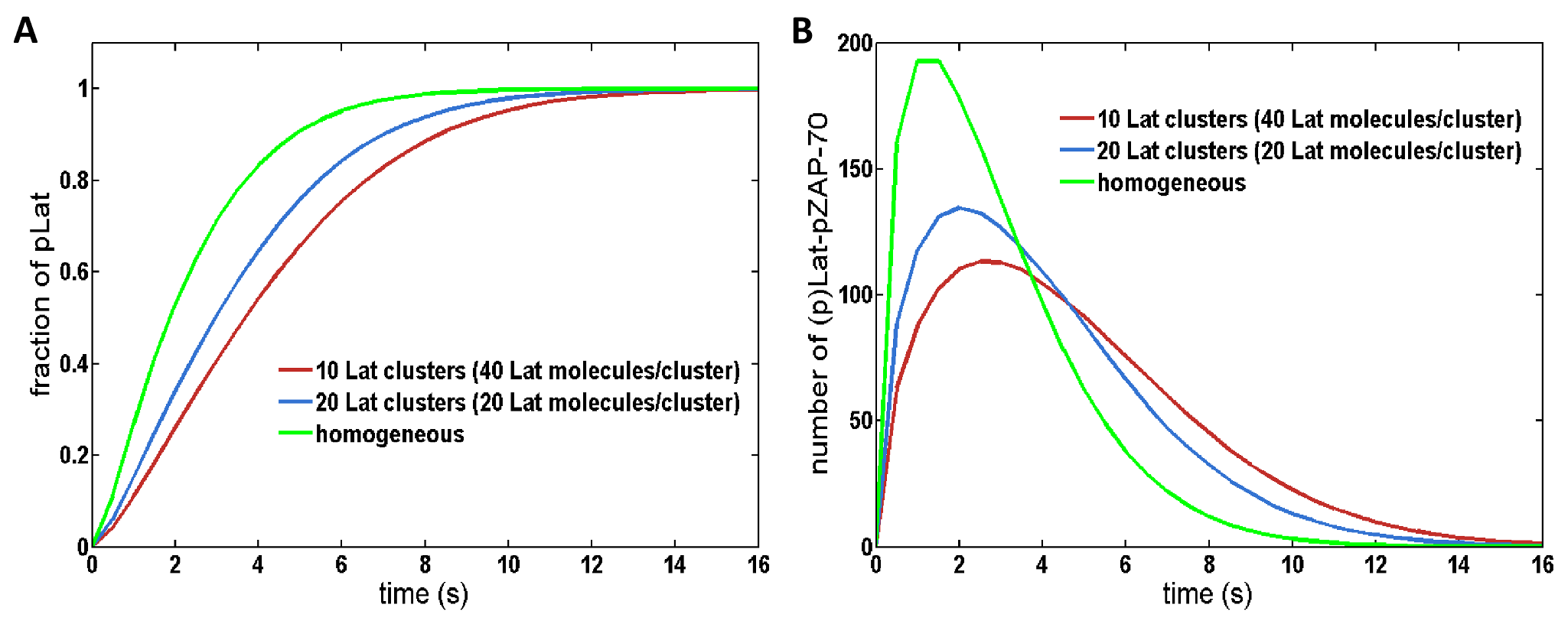

Supplement: Figure S11 — The effect of a decrease in the size of each cluster. The diameter of each cluster is reduced by a factor of 2. (TIF) [file pone.0044444.s011.tif]
