# Supplementary material for: Protein Clusters on the T Cell Surface May Suppress Spurious Early Signaling Events
Source: PLoS One. 2012 Sep 4;7(9):e44444. doi: 10.1371/journal.pone.0044444 (PMC3433417; doi:10.1371/journal.pone.0044444)
Supplement: Table S1 — Rate constants for simulations. (DOCX) [file pone.0044444.s012.docx]

**Table S1.** Rate constants for simulation

| Rate Constants | Reaction |
| --- | --- |
| 0.2 µM^-1^s^-1^ [3] | $k_{on,pZAP70-Lat}$: $pZAP70$binding to $\mathrm{Lat}$ |
| 1 s^-1^ [3] | $k_{off,pZAP70-Lat}$: $pZAP70$ unbinding from $\mathrm{Lat}$ |
| 1 s^-1^ [3] | $k_{p}$: $pZAP70$ phosphorylating $\mathrm{Lat}$ |
| 1 s^-1^ [3] | $k_{off,pZAP70-pLat}$: $pZAP70$ unbinding from $\mathrm{Lat}$ |
| 0.2 µM^-1^s^-1^ | $k_{on,P-pZAP70}$: $pZAP70$ binding to its $\mathrm{phosphatase}, P$ |
| 1 s^-1^ | $k_{off,P-pZAP70}$: $pZAP70$ unbinding from its $\mathrm{phosphatase}, P$ |
| 1 s^-1^ | $k_{dp,pZAP70}$: phosphatase dephosphorylating $pZAP70$ |
| 1 s^-1^ | $k_{off,P-ZAP70}$: $ZAP70$ unbinding from its phosphatase |
| 0.2 µM^-1^s^-1^ | $k_{on,I-pLat}$: $p\mathrm{Lat}$ binding to its $\mathrm{phosphatase}, I$ |
| 1 s^-1^ | $k_{off,I-pLat}$: $p\mathrm{Lat}$ unbinding from its $\mathrm{phosphatas}e, I$ |
| 1 s^-1^ | $k_{dp,pLat}$: phosphatase dephosphorylating $p\mathrm{Lat}$ |
| 1 s^-1^ | $k_{off,I-Lat}$: $\mathrm{Lat}$ unbinding from its phosphatase |
