# Supplementary material for: Protein Clusters on the T Cell Surface May Suppress Spurious Early Signaling Events
Source: PLoS One. 2012 Sep 4;7(9):e44444. doi: 10.1371/journal.pone.0044444 (PMC3433417; doi:10.1371/journal.pone.0044444)
Supplement: Table S2 — Concentration of species for simulations. (DOCX) [file pone.0044444.s013.docx]

**Table S2.** Concentration of species for simulation

| Species | Number of molecules in a 2 µm-diameter membrane system |
| --- | --- |
| Lat  ZAP-70  P  I | 20 per 0.2 µm-diameter cluster (75/ µm^2^ [4], 28.5 per cluster [5])  20 per 0.2 µm-diameter cluster (72000/ µm^3^ [4])  800 (20000/ µm^3^ [6])  800 |
