# Supplementary material for: Protein Clusters on the T Cell Surface May Suppress Spurious Early Signaling Events
Source: PLoS One. 2012 Sep 4;7(9):e44444. doi: 10.1371/journal.pone.0044444 (PMC3433417; doi:10.1371/journal.pone.0044444)
Supplement: Materials and Methods — (DOCX) [file pone.0044444.s014.docx]

**Supporting Information**

**Materials and Methods**

Phosphorylation of Lat

$$pZAP70 + Lat \underset{\to}{k_{on,pZAP70-Lat}}pZAP70-Lat$$

$$pZAP70-Lat \underset{\to}{k_{off,pZAP70-Lat}}pZAP70+ Lat$$

$$pZAP70-Lat \underset{\to}{k_{p}}pZAP70-pLat$$

$$pZAP70-pLat \underset{\to}{k_{off,pZAP70-pLat}}pZAP70+ pLat$$

Dephosphorylation of pZAP-70 by its phosphatase, P

$$P+pZAP70 \underset{\to}{k_{on,P-pZAP70}} P-pZAP70$$

$$P-pZAP70 \underset{\to}{k_{off,P-pZAP70}} P+pZAP70$$

$$P-pZAP70 \underset{\to}{k_{dp,pZAP70}}P-ZAP70$$

$$P-ZAP70\underset{\to}{k_{off,P-ZAP70}} P+ZAP70$$

Dephosphorylation of pLat by its phosphatase, I

$$I+pLat \underset{\to}{k_{on,I-pLat}} I-pLat$$

$$I-pLat \underset{\to}{k_{off,I-pLat}} I+pLat$$

$$I-pLat \underset{\to}{k_{dp,pLat}}I-Lat$$

$$I-Lat\underset{\to}{k_{off,I-Lat}} I+Lat$$

Simulation Methodology

We employed the Stochastic Simulation Compiler [1] to simulate reaction events and diffusion in our system. All reaction rate constants and diffusion constants are converted into units of s^-1^ for use in the simulations [1,2]. First order reaction constants (s^-1^) are unmodified for our simulation. However, experimentally obtained second order rate constants have units of M^-1^s^-1^. To convert into simulation units, we first change the bulk on-rate into an effective two-dimensional on-rate by dividing it by a distance associated with the distance proteins can sample in the direction perpendicular to the membrane (we assume this is of the same order of magnitude as the radius of gyration of molecules). For our simulation, we set the length of a lattice site as 0.01 µm and take the radius of gyration of molecules as 0.001 µm. Then, the experimental value of k_2_ in M^-1^s^-1^ can be converted into s^-1^ by a multiplication factor of 1/60.2 (1 M^-1^s^-1^ =$\frac{1 L}{1 mole\times\sec}=\frac{{10}^{15} {\mu m}^{3}}{6.02\times{10}^{23}\mathrm{molecules}\times\sec}$. After dividing this unit by the square of the length of a lattice site and then by the radius gyration, we obtain 1/60.2 s^-1^). Diffusion is modeled by molecules hopping to nearest neighbor lattice sites. The diffusion coefficient, measured in units of µm^2^/s, is converted into a hopping rate by dividing the by the square of the length of a lattice site.

**Literature Citation**

1. Lis M, Artyomov MN, Devadas S, Chakraborty AK (2009) Efficient stochastic simulation of reaction-diffusion processes via direct compilation. Bioinformatics 25: 2289-2291.

2. Artyomov MN, Lis M, Devadas S, Davis MM, Chakraborty AK (2010) CD4 and CD8 binding to MHC molecules primarily acts to enhance Lck delivery. Proc Natl Acad Sci U S A 107: 16916-16921.

3. Prasad A, Zikherman J, Das J, Roose JP, Weiss A, et al. (2009) Origin of the sharp boundary that discriminates positive and negative selection of thymocytes. Proc Natl Acad Sci U S A 106: 528-533.

4. Das J, Ho M, Zikherman J, Govern C, Yang M, et al. (2009) Digital signaling and hysteresis characterize ras activation in lymphoid cells. Cell 136: 337-351.

5. Williamson DJ, Owen DM, Rossy J, Magenau A, Wehrmann M, et al. (2011) Pre-existing clusters of the adaptor Lat do not participate in early T cell signaling events. Nat Immunol 12: 655-662.

6. Wylie DC, Das J, Chakraborty AK (2007) Sensitivity of T cells to antigen and antagonism emerges from differential regulation of the same molecular signaling module. Proc Natl Acad Sci U S A 104: 5533-5538.
